# Maternal-focused interventions to improve infant growth and nutritional status in low-middle income countries: A systematic review of reviews

**DOI:** 10.1371/journal.pone.0256188

**Published:** 2021-08-18

**Authors:** Victoria von Salmuth, Eilise Brennan, Marko Kerac, Marie McGrath, Severine Frison, Natasha Lelijveld

**Affiliations:** 1 Department of Population Health, London School of Hygiene and Tropical Medicine, London, United Kingdom; 2 Emergency Nutrition Network, Kidlington, Oxford, United Kingdom; 3 Centre for Maternal, Adolescent, Reproductive & Child Health (MARCH), London School of Hygiene & Tropical Medicine, London, United Kingdom; 4 Department of Infectious Disease Epidemiology, London School of Hygiene and Tropical Medicine, London, United Kingdom; Helen Keller International, SIERRA LEONE

## Abstract

**Background:**

Small and nutritionally at-risk infants under 6 months (<6m) are a vulnerable group at increased risk of mortality, morbidity, poor growth and sub-optimal development. Current national and international (World Health Organization) management guidelines focus mainly on infants’ needs, yet growing evidence suggests that maternal factors also influence infant outcomes. We aimed to inform future guidelines by exploring the impacts of maternal-focused interventions on infant feeding and growth.

**Methods:**

We conducted a systematic review of reviews published since 2008 (PROSPERO, register number CRD 42019141724). We explored five databases and a wide variety of maternal-focused interventions based in low- and middle-income countries. Infant outcomes of interest included anthropometric status, birthweight, infant mortality, breastfeeding and complementary feeding practices. Given heterogenous interventions, we present a narrative synthesis of the extracted data.

**Results:**

We included a total of 55 systematic reviews. Numerous maternal interventions were effective in improving infant growth or feeding outcomes. These included breastfeeding promotion, education, support and counselling interventions. Maternal mental health, while under-researched, showed potential to positively impact infant growth. There was also some evidence for a positive impact of: women’s empowerment, m-health technologies, conditional cash transfers, water, sanitation and hygiene and agricultural interventions. Effectiveness was increased when implemented as part of a multi-sectoral program. Antenatal supplementation with macronutrient, multiple micronutrients, Vitamin D, zinc, iron folic acid and possibly calcium, iodine and B12 in deficient women, improved birth outcomes. In contrast, evidence for postnatal supplementation was limited as was evidence directly focusing on small and nutritionally at-risk infants; most reviews focused on the prevention of growth faltering.

**Conclusion:**

Our findings suggest sufficient evidence to justify greater inclusion of mothers in more holistic packages of care for small and nutritionally at-risk infants aged <6m. Context specific approaches are likely needed to support mother-infant dyads and ensure infants survive and thrive.

## Introduction

Malnutrition remains a major global health problem, with approximately 47 million children under five years wasted, and 149 million children still stunted in 2020 [1]. Undernutrition is an underlying cause of 45% of childhood mortality [2]. Recently, there has been an increasing awareness that infants aged under six months (<6m) make up a sizable proportion of wasted children [3], with some 3.8 million infants <6m severely wasted and 4.7 million moderately wasted [4]. An even greater number of infants are “small or nutritionally at-risk” [5]. This term includes infants who are at increased risk of morbidity, mortality or suboptimal development due to any of the following: low birth weight (LBW) due to intra-uterine growth retardation and/or prematurity; anthropometric deficit as used in standard case definitions of undernutrition: low weight-for-age, weight-for-length or length-for-age; postnatal growth faltering even if above the usual -2 Z-score criteria for undernutrition.

Short term risks include infection and mortality which are higher among this age group than in older children [6, 7]. Poor early-life nutrition has also been linked with adverse long-term outcomes, such as impaired cognitive development and higher risk of non-communicable diseases later in life [8–12].

Despite being so vulnerable, the management of small and nutritionally at-risk infants <6m represents a critical care gap, as available evidence often does not address them directly [13]. In 2013, the World Health Organization Guidelines for the Management of Severe Acute Malnutrition included, for the first time, a chapter focused just on infants <6m, however recommendations were based on limited and low quality evidence [14]. Many of the recommended management interventions focused on the clinical and nutritional needs of the infant, yet growing evidence shows a wide range of underlying causes of nutritional vulnerability [15], many of which could be better addressed through maternal-focused interventions, and management of a mother-infant dyad [16–20].

Maternal factors including poor antenatal and postnatal nutrition are well documented as being associated with adverse neonatal and infant outcomes [2, 21–23]. Evidence also suggests that maternal social, environmental, and other physical and mental health factors all also contribute to infant health and growth [16, 18–20, 24]. What is missing is a collation of this evidence to help understand what might best improve future care for small and nutritionally at-risk infants <6m. In this review we thus aim to collate available evidence from existing literature reviews on the impacts of maternal-focused interventions on infant feeding and growth in low- and middle-income countries (LMICs). This will help to inform future research and policymaking.

## Methods

To capture as wide as possible a range of interventions, we conducted a systematic review of reviews. We focused on interventions that impact the immediate and underlying causes of undernutrition as depicted by the UNICEF Conceptual Framework on the Determinants of Maternal and Child Nutrition [25]. We registered our work on the International prospective register of systematic reviews (PROSPERO, register number CRD 42019141724), which we later expanded to also include a search of maternal macro- and micronutrient supplementation (MEDLINE and Cochrane databases only).

### Search strategy

We searched five different databases: MEDLINE, EMBASE, Global Health, Cochrane Library and CINALH plus. We considered reviews published since 2008 (the last 10 years since this project was originally started). The most recent search took place on 29^th^ of October 2020. Reference lists of eligible reviews and personal communications with relevant authors were additional search sources.

Search concepts based around the below PICO were developed for each database. Eligible studies were systematic reviews that included the following:

- **Population (P):** Studies targeting mothers, pregnant women, or women of reproductive age.
- **Intervention (I):** A wide range of intervention types was considered, broadly categorised as: education and counselling; hydration; water, sanitation and hygiene; women’s empowerment; relaxation therapies; multi-sectoral interventions; m-health (i.e. those that use mobile or wireless technologies); conditional cash transfers; agriculture; maternal supplementation during pregnancy; and maternal supplementation during lactation.
- **Control (C):** Though reviews of randomised controlled trials were ideal, we also included reviews which included observational and uncontrolled original studies.
- **Outcomes (O):** We included any review documenting infant growth or nutritional status in the first six months of life, including those aimed at preventing deterioration.

Search terms included: "pregnant women” "mothers", "nutrition- sensitive interventions” "nutrition-specific interventions", "dietary supplementation", "nutritional status", "malnutrition", "feeding practices", "infant mortality", "infant growth", "breastfeeding promotion” "relaxation therapy", and "mental health" (S1 Table).

Search results were limited to human studies from LMICs as defined by the World Bank [26], published since 2008, and with full texts available in English language.

Search results were exported to Endnote software X9 (Clarivate Analytics, Philadelphia, USA) where duplicates were removed. Two researchers independently screening titles and abstracts followed by full texts. Where an updated version of an existing review was available, only the most recent version was included.

### Data extraction and quality assessment

We used a structured form to extract information from each article, including the author, type of publication, type of intervention, setting of the intervention, outcome measures and the reported effectiveness of the interventions. A meta-analysis was not performed due to the diverse nature of the interventions and outcomes reported, instead data was synthesised in a narrative form. Two methods were used to assess the quality of the evidence: 1) the quality of the review methodology and reporting, and 2) the quality of the evidence available, as assessed by the review authors. In order to rank the quality of the review methodology (1), we used the Grades of Recommendation, Assessment, Development and Evaluation (GRADE) Working Group approach according to British Medical Journal Best Practice [27].

## Results

**Fig 1:**
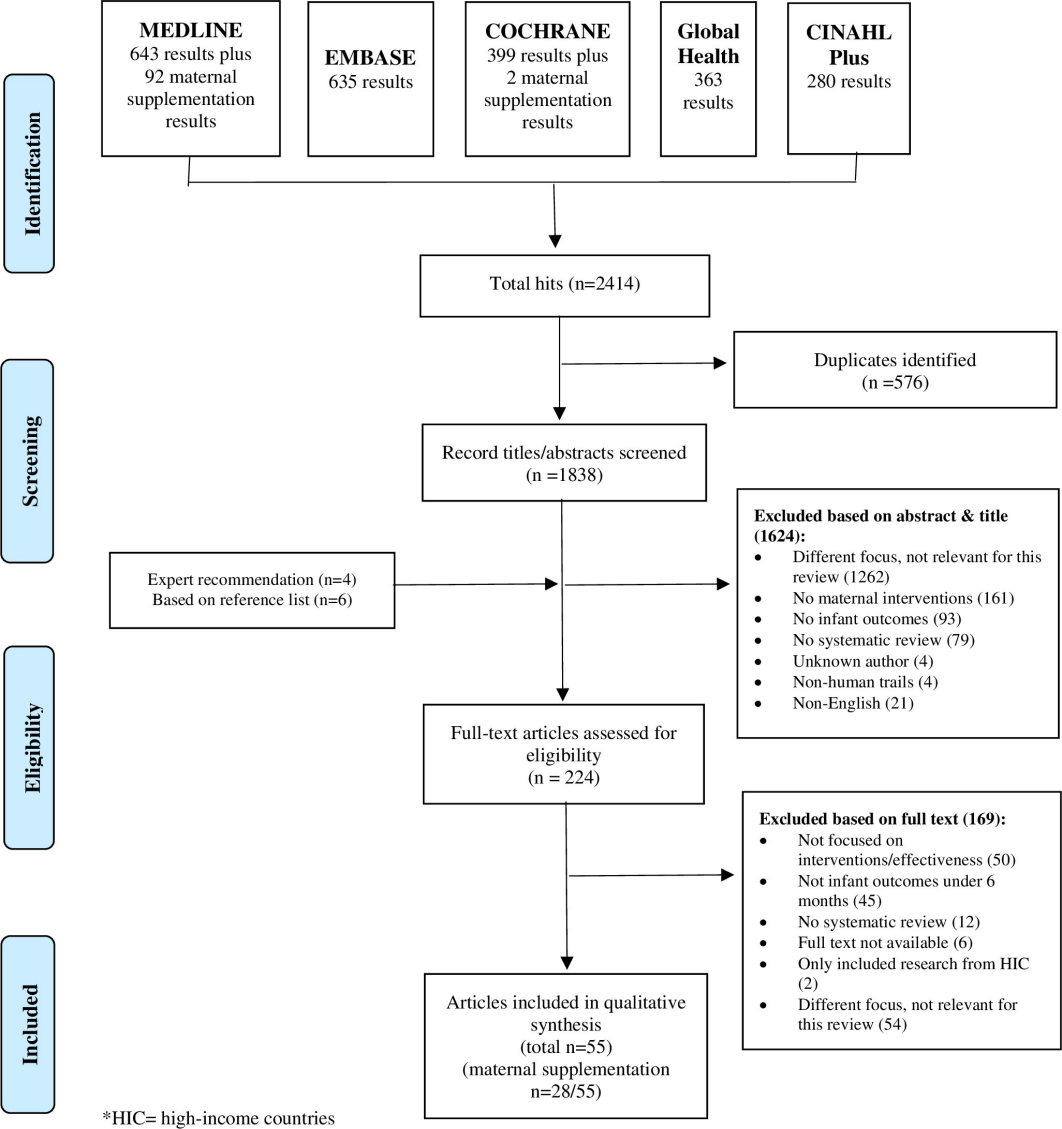
PRISMA flow diagram of search results.

The initial search revealed a total of 2320 reviews, with an additional 94 returned by our search specifically on maternal supplementation (**Fig 1**). We reviewed 224 full texts (214 from the database searches, four recommended by experts via personal communication, and six identified via reference lists) and ultimately identified 55 relevant systematic reviews for inclusion. While all reviews captured studies from LMICs, as per our inclusion criteria, 34 (61%) reviews also incorporated studies from high-income countries (HICs). The studies within the reviews took place across Africa, Asia, The Americas, Europe and Australasia; Asia was the region with the most studies represented. The majority of the evidence related to the prevention of growth faltering, rather than management of existing growth faltering or existing infant undernutrition. Related to this, most studies were aimed at pregnant women rather than during the postnatal period. Many of the reviews also included infants up to 24 months, not just infants <6m. Interventions took place at household-, community- and medical facility level. Three reviews focused their intervention only on adolescent girls [28–30].

We categorised the reviews into the following types of interventions: education and counselling (n = 7); water, sanitation and hygiene (n = 3); women’s empowerment (n = 2); relaxation therapies (n = 1); multi-sectoral interventions (n = 8); m-health (i.e. those that use mobile or wireless technologies) (n = 3); conditional cash transfers (n = 2); agriculture (n = 1); maternal supplementation during pregnancy (n = 23); maternal supplementation during lactation (n = 4); and maternal supplementation during either pregnancy or lactation (n = 1).

### Quality of evidence

The overall quality of the reviews was rated high according to the GRADE evaluation for the majority of reviews (S2 and S3 Tables); only eight (15%) reviews were rated moderate or low. Forty-one (75%) reviews included a meta-analysis. The quality of the studies included in the reviews, however, varied greatly. Most, 48(87%) of the reviews incorporated a structured quality assessment of the primary studies. Low quality evidence was mainly attributed to small sample size, high heterogeneity among study participants, inadequate randomisation, and insufficient blinding in the study set up.

### Maternal-focused interventions

#### Education, counselling and peer support

*Breastfeeding education*, *counselling and peer support*. Three reviews considered the impact of breastfeeding education and counselling interventions. Given the known benefits of breastfeeding for infant health and survival [31], providing breastfeeding support should be a key management and prevention strategy for small and nutritionally at-risk infants <6m. However, Shakya et al. (2017) did not find a sufficient number of eligible studies to assess the effect of community-based peer support on infant nutritional status [32]. Giugliani et al. (2015) and Lassie et al. (2020) also did not find an impact on infant growth outcomes [33, 34]. However, studies largely consisted of healthy infants in HICs, whereas some of the trials that focused on providing breastfeeding education for mothers of LBW infants did find an increase in weight and length [32–34]. Besides growth outcomes, several studies found counselling increased exclusive breastfeeding (EBF) and early initiation of breastfeeding [32–34].

*Other education*, *counselling and peer support*. Twelve reviews evaluated nutritional interventions containing an educational component focusing on topics beyond breastfeeding practices for pregnant women and/or breastfeeding women [28, 34–44]. Five reviews analysed just counselling and education interventions, while seven included counselling/education alongside other interventions e.g. m-health, micronutrient powders, and kangaroo mother care. Most education interventions focusing on the appropriate introduction of complementary feeding were associated with an increase in infants’ weight and length [34, 38] (**Table 1**). A Cochrane review concluded that complementary feeding education may reduce the risk of growth faltering for term-born infants, but modest effects may not be clinically significant, and long-term effects are uncertain [38].

**Table 1:** Summary of maternal non-supplementation interventions during lactation and pregnancy-reviews.

Study	Intervention	Outcome measures	Summary of findings (separated into prevention and management)	Review quality	Reported quality of primary evidence**
***Education and counselling and support groups***					
***Giugliani et al. (2015)* [33].**	Breastfeeding promotion interventions	Weight, length/height z scores, body mass index, WHZ	Prevention**: Non-significant increase in mean weight z score (MD 0.03, -0.06 to 0.12), and mean length/height z scores (MD 0.03, 95% CI: -0.02 to 0.08); mean weight z score, negative effect in low income countries (MD -0.11, CI -0.20 to -0.02), but non-significant positive effect in middle income countries (MD 0.11, CI -0.01 to 0.22).	High-moderate	High-quality evidence (26%); very low/low risk of bias (46%); high risk of bias (29%)
			Management***: RCT for mothers with LBW infants–Increase weight (3620±229 g vs 3315±301 g, P<0.001) and length (50.2±1.3 cm vs 48.7±1.6 cm, P<0.001) at 30-, 45- and 60-days vs control.		
**Shakya et al. (2017) [32]**	Breastfeeding support: Community based one-on-one and/or group peer support	Underweight, stunting, wasting, child feeding practices (birth-6 months)	**Prevention:** Insufficient number of studies to analyse impact on child nutritional status or feeding outcomes; focus on EBF initiation, EBF at 3 months.	High	**41 RCTs/quasi-experimental**: high risk of bias for blinding participants, personal and outcome assessment
			**Management:** An RCT found low-weight babies and malnourished children (under 5 years) who were receiving home visits by mentor mothers were rehabilitated in shorter periods of time compared to those in the control group.		
					**6 observational studies**: 30% of studies had high risk for incomplete data, blinding of assessment and measurement of exposure and selective reporting
***Lassi et al. (2020) [34]***	Breastfeeding and complementary feeding education	EIBF, EBF, HAZ, WAZ, WHZ, neonatal mortality, infant mortality	**Prevention: *Breastfeeding education*—**Positive impact on early initiation (RR 1.20, CI 1.12 to 1.28); no impact on HAZ (MD 0.10, CI -0.04 to 0.25), WAZ (MD -0.04, CI -0.12 to 0.05), WHZ (MD 0.01, CI -0.07 to 0.09), neonatal mortality (RR 1.10, CI 0.90 to 1.24) or infant mortality (RR 0.86, CI 0.73 to 1.02).	High	**Overall**: very low (13.3%), low (50%), moderate (23.3%) and high (13.3%) quality
					**Breastfeeding**: very low-low quality
			***Complementary feeding education–***Food secure settings impact on WAZ (MD 0.41, CI 0.07 to 0.75) and HAZ (MD 0.29, CI 0.04 to 0.54) and in food insecure impact on WAZ (MD 0.47, CI 0.35 to 0.59), HAZ (MD 0.25, CI 0.09 to 0.41) and WHZ (MD 0.50, CI 0.35 to 0.65).		
			**Management: Breastfeeding education–** 2 studies reported on LBW infant outcomes, one study found an increase in weight (3620±229 g vs 3315±301 g, P<0.001) and length after 2 months (50.2±1.3 cm vs 48.7±1.6 cm, P<0.001), the other found no effect on infant weight, length, head circumference at 6 months.		
***Jonmahamed et al. (2020) [35]***	Community home visits and mother/peer groups promoting child health, nutrition and feeding practices.	EIBF, EBF, minimum dietary diversity, minimum meal frequency, stunting, underweight and wasting	**Prevention:** Meta-analysis of RCTs of home visits increased odds of EIBF (OR 1.5, CI 1.12 to 1.99) and EBF (OR 4.42, CI 2.28 to 8.56). Mother/peer groups had no significant effect on EIBF and EBF. Combined intervention increased odds of EIBF (OR 2.13, CI 1.12 to 4.05) EBF (OR 2.43, CI 1.70 to 3.46) and wasting (OR 0.77, CI 0.67 to 0.89), but no effect on stunting and underweight.	High	**Overall:** very low (17.6%), low (58.8%), moderate (17.6%) and high (5.9%) quality
***Arikpo.et al. (2018) [36]***	Educational intervention on the introduction of complementary feeding	Weight, height/length, MUAC, WAZ, height/length age z score, weight for height/length z score, morbidity, mortality	**Prevention:** Little evidence for growth patterns—at 6 months non-significant increased weight 0.03 kg (CI -0.10 to 0.17 higher) and mean height/length 0.16 cm (CI: -0.21 to 0.52); no data on infant mortality; Improvement of complementary feeding; only community intervention increased duration of EBF (RR 2.32, CI 1.45 to 3.73).	High	**Overall:** moderate to very low quality
***Lassi et al. (2019) [37]***	Community-based health education care packages	Neonatal mortality, perinatal mortality, neonatal infections, and health behaviours.	**Prevention:** Reduced overall neonatal mortality (RR 0.87, CI 0.78 to 0.96), early neonatal mortality (RR 0.74, CI 0.66–0.84), late neonatal mortality (RR 0.54, CI 0.40 to 0.74) and perinatal mortality (RR 0.83, CI 0.75 to 0.91); positive effect timely initiation of breastfeeding (RR 1.56, CI 1.37 to 1.77).	High	**Overall:** very low-low
***Ojha et al. (2020) [38]***	Nutrition education interventions for infant weaning	WAZ, HAZ, child growth and neuro-development	Increased WAZ (MD 0.15, CI 0.07 to 0.22) and HAZ (MD 0.12, CI 0.05 to 0.19) by 12 months of age. Uncertain effects on long-term growth and neuro-development outcomes.	High	**Overall:** low-moderate
***WASH***					
***Dangour et al. (2013) [45]***	Water intervention, coverage / use sanitation facilities, handwashing w/soap	WHZ, WAZ, or HAZ	**Prevention:** No effect on WAZ (MD 0.05, 95% CI -0.01 to 0.12), WHZ (MD 0.02, 95% CI -0.07 to 0.11). Small effect HAZ (MD 0.08, 95% CI 0.00 to 0.16).	High	**Overall**: low quality
***Gera et al. (2018) [46]***	Hygiene promotion, education, water intervention, sanitation improvement	Weight, height, WHZ, MUAC, prevalence malnutrition, non-diarrheal morbidity, infant mortality	**Prevention: *Overall*—**Lower risk of underweight (RR 0.81, 95% CI 0.69 to 0.96), stunting (RR 0.77, 95% CI 0.68 to 0.86), wasting (RR 0.12, CI 0.02 to 0.85). Reduced mortality (RR 0.65, 95% CI 0.25 to 1.70).	Moderate	Majority very low quality
			***Improvement in water supply/quality*—**decreased risk of stunting by 13% (RR 0.87, 95% CI 0.81 to 0.94), no effect WAZ (MD 0.03, 95% CI 0.00 to 0.06), reduced mortality (RR 0.45, 95% CI 0.25 to 0.81).		
			***combined WASH*—**improved HAZ (MD 0.22, 95% CI 0.12 to 0.32).		
***Gizaw et al. (2019) [47]***	Water treatment, hand washing, hygiene education, home based water treatment, latrine construction/promotion	Child nutritional status, HAZ	**Prevention:** Increased mean HAZ (SMD 0.14, 95% CI = 0.09 to 0.19), better effect of combined interventions on child growth (SMD 0 0.15, 95% CI = 0.09 to 0.20), pooled effect mean HAZ (SMD 0.19, CI 0.15 to 0.23).	High	Most studies had low risk of bias
***Women’s empowerment***					
***Kraft et al. (2014) [29]***	Gender accommodating-/ transformative, Behaviour Change Communication	Infant mortality, stunting	**Prevention:** Reduction stunting among children > 24 months, nutrition and empowerment combination most effective together.	Moderate-low	Not formally assessed, only included studies with moderate-to-strong evaluation designs
***Taukobong et al. (2016) [48]***	Gender empowerment, family planning, health, nutrition, agriculture, WASH, financial services	Stunting, wasting, underweight, body mass index, MUAC, length/age weight/age, weight/length	**Prevention:** Higher levels of decision-making power improved nutritional status in children + reduced risk of stunting.	Moderate	Not formally assessed, reported that strength of design and analyses varied; limited RCT’s and quasi-experimental studies
**Relaxation therapy**					
***Shukri et al. (2018) [49]***	Relaxation therapy	Infant dietary intake, weight gain, body mass index, breastfeeding (breast milk volume/ milk yield)	**Prevention:** No studies reported on infant growth/behaviours; reduction on maternal stress, evidence for increasing milk yield and fat content.	High-moderate	All studies had limitations regarding design or sample collection procedures–(2/5) not randomised, studies unable to blind participates, many had small sample sizes, did not consider all potential confounders
			**Management:** Strongest evidence from increasing milk yields, and possible fat content in mothers of preterm infants.		
**Multisectoral interventions**					
***Bhutta et al. (2013) [28]***	MMN, folic acid, iron/iron folic acid, calcium, iodine, vitamin D, zinc and balanced energy and protein supplementation, nutrition education, WASH interventions, deworming, IPTp/ITN for malaria in pregnancy, mental health support.	Growth, stunting wasting, mortality, breastfeeding, complementary feeding practices (6–24 months)	**Prevention: *MMN*—**significant effects on LBW (RR 0.88, 95% CI 0.85 to 0.91), SGA (RR 0.89, 95% CI 0.83 to 0.96), preterm birth (RR 0.97, 95% CI 0.94 to 0.99).	High-moderate	Not reported, however, only captured reviews with good quality methods for all interventions.
			***Folic acid—***mean birth weight (MD 135.75g, CI 47.85–223.68g).		
			***Iron/iron folic acid*—**reduction in LBW (RR 0.81, CI 0.68 to 0.97).		
			***Calcium*—**population with low intake, increase birth weight (85g, CI 37 to 133g) and reduction in preterm birth (RR 0.97, CI 0.94 to 0.99).		
			***Iodine—***insufficient data to draw conclusions on mortality, children physical and mental development.		
			***Vit D -***Lack of evidence for functional pregnancy outcomes.		
			***Zinc—***Evidence for reduction in preterm birth, but insufficient evidence for routine supplementation.		
			***Balanced energy/protein supplementation*—**reduction in SGA (RR 0.66, CI 0.49 to 0.89), still births (RR 0.62, CI 0.40 to 0.98) and small increase in birth weight (MD 73g, CI 116 to 130g).		
			***Nutrition education food secure populations*—**height gain (SMD 0.35, 95% CI 0.08 to 0.62), HAZ (SMD 0.22, 95% CI 0.01 to 0.43). Complementary feeding w/without education HAZ (SMD 0.39, 95% CI 0.05 to 0.73), education ***food insecure*** populations: HAZ (SMD 0.25, 95% CI 0.09 to 0.42), stunting (RR: 0.68, 95% CI 0.60 to 0.76).		
			***WASH*—**20% reduction open defecation—0.1‐SD increase height.		
			***IPTp/INT*—**reduction in low birth weight.		
			**Mental health—**promising effect of cognitive behaviour theory during pregnancy on depression post-partum, no evidence effect on infant weight or linear growth.		
***Goudet et al. (2019) [39]***	Zinc, micronutrient and macro nutrition supplementation and nutrition education.	length-for-age/HAZ birth weight, LBW	**Prevention: *Zinc*–**no effect on new-born length (MD -0.13, CI -0.36 to 0.10), LBW (MD -36.2, CI -83.61 to 11.35).	High	**Overall**: Low-moderate quality
			***Micro or macro nutrient supplementation-*** unclear effect for length-for-age/HAZ.		
			***Education***- Positive impact on LBW (MD 478.4g, 95% CI 423.55 to 533.32g).		
***Ruel et al. (2013) [30]***	Agriculture, Social safety nets, Early child development	Maternal and child nutritional status	**Prevention:** Community-based peer support is effective in increasing EBF duration among mothers. Targeted agriculture interventions have an important role in supporting healthy diets, but inconclusive evidence for the impact on children’s nutritional status, with the possible exception of Vit A intake and status.	Moderate	Not formally assessed, however, reported that studies often suffered from poor quality impact evaluations
***Victora et al. (2012) [40]***	Large scale iron folic acid, food supplementation/ nutrition education, counselling/ + conditional cash transfers programmes	Birth weight, maternal weight gain, caregiver knowledge, behavioural change, dietary habits, anaemia	**Prevention: *iron folic acid***: Positive impact on the prevalence of maternal anaemia.	Low	Not formally assessed, only large scale programs that were adequately evaluated were utilised, high risk of publication bias for grey lit
			***Food supplementation/ nutrition education + counselling*:** improved caregiver knowledge and behaviour change and improving utilisation of antenatal care, dietary habits, no effect on maternal weight gain, effect on birth weight unclear.		
			**+ *conditional cash transfers*:** some evidence for positive impact on birth weight.		
***Park et al. (2019) [41]***	Micronutrient supplementation, lipid-based supplementation or food supplementation, deworming, maternal education, and WASH.	Preterm birth, birth weight, length-for-age z score	**Prevention:** MMN reduced preterm birth (OR 0.54, CI 0.27 to 0.97), while iron, iron plus calcium, calcium plus vitamin D, zinc, iron folic acid plus vitamin A, MMN, a single dose of deworming plus iron and 118 kcal of Lipid-based nutrient supplementation, showed modest improvements in birth weight.	High	*Most studies had low/unclear risk of bias, apart from performance bias, where 23.1% of studies had a high risk of bias due participants not being blinded to intervention group*
			No intervention for mothers with infants 0-6months led to statistically significant improvements in length-for-age z score.		
***Park et al. (2020) [42]***	MMN, iron folic acid, Vit D lipid based nutrient supplements, deworming, WASH, food provision and maternal education	Length-for-age z score	**Prevention:** No statistically significant effect of MMN, Vit D, iron folic acid supplementation for lactating mothers, food supplements, maternal education, deworming or WASH on length-for-age z score.	High	*Majority of studies had low/unclear risk of bias, but high risk of performance bias (34% high risk) due to lack of blinding participants*
***Ota et al. (2015) [43]***	Nutrition education and balanced energy and protein supplementation	Preterm, birth weight, neonatal death, child growth, still birth, neurological development	**Prevention: *Nutritional education*—**Decrease risk preterm (RR 0.46, 95% CI 0.21 to 0.98), LBW (RR 0.04, CI 0.01 to 0.14), increased birth weight undernourished women (MD 489.76 g, CI 427.9- to 551.6).	High	Majority low-moderate quality
			***Balanced energy and protein supplementation***–positive impact on stillbirths (RR 0.60, CI 0.39 to 0.94), mean birth weight (MD 40g, CI 4.66 to 77.26), and SGA (RR 0.79, CI 0.69 to 0.90), no effect on preterm birth.		
			**High protein supplementation—**increased risk of SGA (RR 1.58, CI 1.03 to 2.41).		
***East et al. (2019) [44]***	Social support via home visits, antenatal clinic visits, telephone or combination for women at increased risk of LBW babies	Birth weight, stillbirth/neonatal death, gestational age, infant morbidity, postnatal depression	**Prevention:** Overall slight positive impact on postnatal depression, may have a slight impact on birth weight (RR 0.94, CI 0.86–1.04) and gestational age (RR 0.92, CI 0.84 to 1.01); no impact on stillbirth/neonatal death; positive impact on caesarean sections (RR 0.90, CI 0.83–0.97) and number of antenatal hospital admissions (RR 0.78, CI 0.68 to 0.91).	High	Overall: moderate quality
***m-Health***					
***Saronga et al. (2019) [50]***	Interventions using mobile Health technologies	Birth weight, growth, breastfeeding initiation/duration; pregnancy weight gain, maternal knowledge	**Prevention**: LBW vs control group (30% vs. 35%); No other infant nutritional outcomes reported. Maternal weight gain improved.	High-moderate	Only 4 studies captured but all studies were positive/neutral quality
***Palmer et al. (2020) [51]***	Targeted client communication via mobile phones.	Health behaviour change, service utilisation e.g. antenatal care, maternal morbidity/mortality, maternal morbidity (mental), neonatal health, partner violence, well-being, acceptability.	**Prevention:** In settings where breastfeeding is less common, women receiving targeted client communication via mobile may breastfeed more, when compared to standard care.	High	**Overall:** very low-low quality
			*Targeted client communication via mobile devices effect on women’s and infant’s morbidity and mortality when compared to standard care is unclear as evidence is of very low certainty*.		
***Sondaal et al. (2016) [52]***	Unidirectional text/voice messaging, direct two-way communication, multidirectional text messages, unidirectional telephone counselling	Maternal and neonatal service utilisation, maternal health, mortality and morbidity, perinatal mortality, maternal knowledge.	***Prevention*:** No consistent effects of mHealth interventions on maternal and neonatal health outcomes were observed; however, single studies found positive trends in reducing perinatal mortality and improving breastfeeding practices.	High-moderate	**Overall:** moderate quality
			Inconclusive findings were observed for mHealth effects on maternal knowledge but positive effects on service utilisation.		
**Conditional cash transfers**					
***Leroy et al. (2009) [53]***	Conditional cash transfers with or without micronutrient- fortified food, health nutritional education	Height, HAZ, weight, weight-for length, WAZ, child micronutrient status	**Prevention:** Small effect on the elimination of linear growth retardation, small impact on micronutrient nutrition status.	Moderate-low	Not formally assessed, however reported low publication bias
***Lagarde et al. (2009) [54]***	Conditional cash transfers, to households +/- nutrition supplementation	Weight, growth, height, HAZ, WAZ children <24 months, stunting, underweight	**Prevention:** Increase in height of 1cm amongst children < 4 y; impact on HAZ mixed results (significant increase while another one found a negative impact); Decrease probability of being stunted, underweight or chronically malnourished.	High	Moderate quality for all outcome apart from health service utilisation (low quality)
**Agriculture**					
***Masset et al. (2012) [55]***	Bio-fortification, home gardens, small scale fisheries/ aquaculture, dairy development	Height-for-age, weight-for-age, weight-for-height, prevalence stunting, wasting, underweight, micronutrient intake	**Prevention:** Out of 8 studies, only one found a small but statistically significant effect on stunting; however, out of seven studies, three and two studies found a positive statistically significant effect on underweight and wasting, respectively.	Moderate	Studies were not of high quality, high overall risk of bias; bias introduced due to the low number of RCT’s, high selection bias, small samples sizes, lack of power calculations and outcomes measurements.

Height-for-age z score (HAZ); weight-for-age z score (WAZ); mid-upper arm circumference (MUAC); small for gestational age (SGA); Mean difference (MD); Relative risk (RR); low birth weight (LBW); Randomised control trials (RCTs); multiple micronutrients (MMN); Water sanitation and hygiene (WASH); intermittent prevention treatment in pregnancy/insecticide treated bed nets (IPTp/ITN); weight-for-height z scores (WHZ); Early initiation of breastfeeding (EIBF); Exclusive breastfeeding (EBF): Odds ratio (OR); Standard mean difference (SMD).

*Quality reported in the respective reviews

** Prevention of growth faltering in infants <6m

***Management of small and nutritionally at-risk infants <6m.

Community health worker home visits combined with mother/peer support groups showed a positive effect on reducing the risk of wasting, but no effect on stunting or underweight [35, 42]. Reviews of antenatal nutrition education programs have found mixed effects, with some suggesting they can improve infant birth weight and reduce risk of preterm birth, but others showed no or limited effect [39–44]. Community health education programmes were also shown to reduce neonatal mortality, especially when provided during both antenatal and postnatal periods, and when additional family members are incorporated [37]. However, a Cochrane review of social support programs (home visit, regular antenatal care) for mothers at increased risk of LBW babies concluded that they are unlikely to have a large impact on the proportion of LBW and preterm births [44].

#### Water, sanitation and hygiene (WASH)

The effectiveness of WASH interventions on infant outcomes are mixed [28, 41, 42, 45–47]. A Cochrane review including 14 studies found that an increase in height was more responsive to WASH interventions in children under 24 months of age, whereas increase in weight was more responsive in children 25‐60 months of age [45]. Overall, combined WASH interventions were found to be more effective than single interventions in improving infant outcomes [45–47]. Only a small selection of possible WASH interventions was applied within the studies, with poor quality assessment and relatively limited duration [45–47].

#### Women’s empowerment

Women’s empowerment has been hypothesised to improve children’s nutritional outcomes by enabling women to use and realign resources and household practices to enhance their children’s nutritional well-being. Related interventions were assessed in two different reviews [29, 48]. The three most important gender-related levers were identified as being: control over income/assets/resources, decision-making power and education [48]. Higher levels of decision-making power were associated with improved nutritional status in children and a reduced risk of stunting [48]. Interventions targeting both men and women, however, showed mixed results on effectiveness, while adolescent- specific interventions had no effect on infant health outcomes [29].

#### Relaxation therapy

There are a small but growing number of studies on relaxation therapy, the core idea being that relaxation (via a range of options including music and guided breathing exercises) could be an easy, quick and deliverable intervention with a clear and direct biological pathway to improved infant growth via increased breastmilk supply. One systematic review was identified. Though it found no studies reporting on infant outcomes, it did find that maternal relaxation was associated with reduced maternal stress, increased milk yield and milk fat levels, especially in mothers with preterm infants [49].

#### m-Health

Three reviews focused on m-health interventions [50–52]. One found that LBW was less common among women in the intervention group (short message service, audio voice messages or a combination) compared with the control group [50]. M-Health was also found to be useful in encouraging consumption of micronutrient supplements in pregnancy [50]. Targeted messaging via mobile devices was found to marginally improve breastfeeding rates, but had little or no effect when compared to non-digital strategies [51]. Few studies within the reviews presented growth and mortality outcomes, but one study, which combined educational text messages and two-way voice calls found a significant reduction in perinatal mortality [52].

#### Conditional cash transfer programs

Three reviews assessed the impact of conditional cash transfers on infant outcomes. However, these reviews were mostly based on programs implemented in Latin and Central America and referred to the same study populations [40, 53, 54]. The programs generally combined basic cash transfer with health and nutrition education [53]. An overall positive effect of conditional cash transfers programs on the risk of stunting and underweight was reported [54] alongside a possible impact on birth weight [40]. There was a greater impact of conditional cash transfers on younger children (infants <6m vs children 6–48 months), children from lower socio-economic backgrounds and children with longer exposure to the intervention [53, 54]. Studies also found improvements in maternal micronutrient consumption but also consumption of high-fat foods [53, 54].

#### Maternal mental health

While there are strong associations between maternal mental health and infant outcomes in observational work, intervention studies are still lacking. One review included maternal mental health interventions alongside other interventions. They report that cognitive behaviour therapy during pregnancy had no impact on infant weight or growth, but a positive impact on depression post-partum [28]. A Cochrane review of social support programs for mothers at increased risk of LBW babies found a slight positive impact on postnatal depression [44].

#### Agriculture interventions

Agricultural interventions such as bio-fortification, home gardens, small scale fisheries and aquaculture, dairy development, animal husbandry and poultry development rarely reported the effect on child nutritional outcomes [55]. A limited amount of studies reported a positive impact on Vitamin A status, stunting, underweight and wasting [30, 55]; however, studies often lacked high-quality impact evaluations. The effect of agricultural interventions appeared to be bigger when targeting women within empowerment or educational activities to foster their knowledge and skills and increase control over income [30].

#### Multi-sector interventions

Given that the determinants of infant growth are multi-faceted, eight reviews summarised the evidence base for multiple interventions across sectors [28, 30, 39–44]. Many of them combined micronutrient supplementation with antenatal education or counselling, however WASH interventions, mental health and social support, agriculture and social safety nets were also captured. Scaling up of nutrition packages that include optimising infant feeding and increasing maternal dietary diversity during pregnancy may successfully reduce the risk for stunting and wasting in children under five years [28]. Implementing a range of multisectoral actions/interventions rather than focusing on one particular intervention appears to increase program effectiveness [28, 30]. However, more research is needed as interventions are still often implemented and evaluated in isolation [41]. Furthermore, more comprehensive evaluation tools are needed, to avoid dismissing an effective program due to a lack of effect on a small set of outcomes [40].

#### Maternal supplementation during pregnancy

**Of the 28 reviews focused on maternal supplementation and infant outcomes, most, 23 (82%) review the evidence of supplementation in pregnancy (Table 2). Overall, the reviews found that food fortification** and **macronutrient interventions had a positive effect on b**irth weight and length, risk of stillbirth and small for gestational age (SGA) [28, 41, 43, 56–60] though there was no evidence of longer-term benefits for child growth and development [57]. A recent Cochrane review on **lipid-based nutrient supplements in pregnancy found positive impacts on birth weight, length and** SGA when compared to iron folic acid supplementation, but no benefit compared to multiple micronutrient (MMN) supplementation [61].

**Table 2:** Summary of maternal supplementation interventions, during lactation and pregnancy–reviews.

Study	Intervention	Outcome measures	Outcomes article	Review quality	Reported quality of primary evidence*
***Pregnancy***					
***Gresham et al. (2014) [56]***	Food and fortified food product, macronutrient interventions, counselling plus food	Birth weight, LBW, weight, SGA, head circumference macrosomia, large for gestational age, perinatal mortality	**Overall:** Dietary interventions targeted at-risk populations positive impact birth weight (SMD 0.26, CI 0.09 to 0.41) and LBW (SMD -0.19, CI -0.37 to -0.02).	High	Positive (61%) and neutral (39%) quality
			**Food and fortified products:** impacted birth weight (SMD 0.27 CI 0.14 to 0.40), the incidence of LBW (SMD -0.22, CI -0.37 to -0.06).		
			**Macronutrient interventions:** impacted length (SMD 0.07, CI 0.01 to 0.14) and birth weight (SMD 0.23, CI 0.07 to 0.38) and incidence LBW (SMD -0.19, CI -0.34 to -0.04).		
***Visser et al. (2018) [57]***	Food distribution and supplementary feeding programmes	SGA, stillbirth, birth weight, birth length, height gain, weight gain, neonatal death	Impacted stillbirth (RR 0.60, CI 0.39 to 0.94), infant birth weight (MD 40.96, CI 4.66 to 77.26), SGA, birth length (MD 0.16 CI 0.10 to 0.31). No long-term benefits for the child in terms of growth and neurocognitive development.	High	Very low-moderate quality
			High protein supplementation was associated with increased risk of SGA.		
***Stevens et al. (2015) [58]***	Balanced protein energy supplementation in undernourished women	Birth weight, birth length, head circumference, physical growth	Impacted birth weight (MD 0.20g, CI 0.03 to 0.38g).	High	Low (28.2%), moderate (43.6%) and high (28.2%) quality
			No significant effect on birth length and head circumference.		
			Impact on long-term growth is inconclusive–One RCT which showed a significant difference in height till 60 months and weight until 24 months.		
***Imdad et al. (2011) [59]***	Balanced protein energy supplementation	SGA, neonatal mortality, birth weight	Impact SGA (RR 0.69, CI 0.56 to 0.85), birth weight (MD 59.89g, CI 33.09 to 86.86g), effect was more pronounced in malnourished women.	High	Overall moderate quality
			No impact on neonatal mortality.		
***Pimpin et al. (2019) [60]***	Protein from animal-sourced foods	Birth weight, LBW, SGA, height, and length	Impacted birth weight (MD 0.06kg, CI 0.02 to 0.11kg) and maternal weight gain. No impact LBW, SGA, height or weight in later childhood.	High	Low (34%), moderate (255) and high (41%) quality
***Ramakrishnan et al. (2012) [62]***	MMN supplementation	LWB, birth weight, SGA, gestational age, preterm birth, stillbirth, neonatal death, maternal mortality	Impacted LBW (RR 0.86, CI 0.81 to 0.91), birthweight (MD 52.6g, CI 43.18 to 62.03g), SGA RR 0.83, CI 0.73 to 0.95), gestational age (WMD 0.08 weeks, CI 0.01 to 0.14 weeks).	High	Overall moderate-high quality
			No impact preterm, stillbirth, neonatal death, maternal mortality; increased risk of neonatal death when compared with iron–folate in the subgroup of five trials that began after the first trimester (RR 1.38, CI 1.05 to 1.81). No impact maternal mortality (RR 0.96, CI 0.64 to 1.45).		
***Thorne-Lyman et al. (2012) [63]***	Vitamin A and carotenoids	LBW, SGA, birth weight, weight gain in pregnancy, preterm birth, gestational age, neonatal mortality, morbidity, infant and child growth, mortality and morbidity, maternal mortality, pre-eclampsia, maternal anaemia	No impact on SGA and birth weight, preterm, stillbirth foetal death, maternal mortality, morbidity or birth complications, infants and neonatal mortality; no impact of mother-to-child HIV transmission in a pooled analysis, although some evidence suggests that it may increase transmission.	High-moderate	Overall low-moderate quality; very low (10%), low (45%), moderate (21%) and high (24%) quality
			Impact on LBW for HIV+ populations (RR 0.79, CI 0.64 to 0.99).		
			Little evidence of an effect on WAZ and HAZ.		
			Improved haemoglobin levels and risk of anaemia (RR 0.81, CI 0.69 to 0.94)		
***Das et al. (2018) [61]***	Lipid-based nutrient supplement	Maternal anthropometric status, maternal mortality, adverse maternal outcomes, LBW, birth weight, birth length, SGA, preterm, child development, duration of gestation, stillbirths, head circumference, infant mortality, neonatal mortality, MUAC, stunting, wasting and underweight in the first 6 months.	**compared iron folic acid:** impacted birth weight (MD 53.28g, CI 28.22 to 78.33), birth length (MD 0.24cm, CI 0.11 to 0.36), SGA (RR 0.94, CI 0.89 to 0.99), length of gestation (MD 0.18weeks, CI 0.04 to 0.32), stunting (RR 0.82, CI 0.71 to 0.94); Insufficient data of child development and no data on wasting and infant mortality	High	Overall moderate quality
			**compared to MMN**: No data stillbirth, wasting and infant mortality, no impact maternal, infant and birth outcomes.		
***Middleton et al. (2018) [64]***	Omega-3 fatty acids	Preterm, gestation, perinatal deaths, LBW, Large for gestational age, SGA, intrauterine growth restrictions, adverse maternal effects, child neurodevelopment and growth, adult body mass index	Positive impact on preterm birth (RR 0.89, CI 0.81 to 0.97), perinatal deaths (RR 0.75, CI 0.54 to 1.03) and LBW (RR 0.90, CI 0.82 to 0.99); Small increase large for gestational age (RR 1.15, CI 0.97 to 1.36), little/no difference in SGA or intrauterine growth restrictions.	High	Very low-moderate quality
			Little difference in child neurodevelopment and growth outcomes		
			insufficient evidence to determine effects on maternal adverse events and postnatal depression		
***Harding et al. (2017) [65]***	Iodine	Perinatal mortality, LBW, preterm birth, SGA, infant/child physical and mental development, mortality, Impacted postpartum hyperthyroidism and digestive intolerance in pregnancy	Positive impact perinatal mortality (RR 0.66, CI 0.42 to 1.03), congenital anomalies (RR 0.27, CI 0.12 to 0.60), uncertain data on neonatal mental/motor development but higher child mental development score (MD 11.21, CI 7.97 to 14.46)	High	Overall very low-low quality
			Impacted postpartum hyperthyroidism (RR 0.32, CI 0.11 to 0.91) and digestive intolerance in pregnancy (RR 15.33, CI 2.07 to 113.7)		
			No trials reported on infant/child growth or infant death; No impact LBW, preterm or SGA; All data from regions with mild to moderate iodine deficiency.		
**Farebrother at al. (2018) [66]**	Iodine	Birth length, weight, and head circumference	**Overall:** no impact on birth length, weight or head circumference: **severely deficient women:** positive impact on head circumference and birth weight (MD 200g, CI 183 to 217g).	High	Overall very low quality
***Zhou et al. (2013) [67]***	Iodine	Child development and growth	***Regions severe deficiency***: *reduced risk of cretinism, but no impact on childhood intelligence, gross development, growth, or pregnancy outcomes, although evidence of improvement in some motor functions*.	High-moderate	None of the studies reported adequate random-sequence generation, high risk of bias due to incomplete data, unclear risk for blinding due to inadequate reporting
			***Regions mild-moderate deficiency***: *No data on child growth and development*.		
***Rumbold et al. (2015) [68]***	Vitamin E	Preeclampsia, preterm, stillbirth, neonatal death, perinatal death, intrauterine growth restrictions, maternal death, maternal morbidity, infant death, gestational age, child growth, congenital malformations, Apgar score	No impact stillbirth, neonatal death, infant death, preterm, pre-eclampsia, intrauterine growth restrictions, birth weight, maternal death, gestational age, congenital malformations, Apgar score, maternal morbidity/adverse effects.	High	Majority moderate-high quality but low for preterm PROM
			Increased risk self‐reported abdominal pain (RR 1.66, CI 1.16 to 2.37) and term prelabour rupture of membranes (PROM) (RR 1.77, CI 1.37 to 2.28); no corresponding increased risk for preterm PROM. No data on child growth.		
***Rumbold et al. (2015) [69]***	Vitamin C	Stillbirth, neonatal death, perinatal death, intrauterine growth retardation, preterm, maternal death, infant death, gestational age, birth weight, congenital malformations, poor childhood growth, PROM, eclampsia	No impact on stillbirth, neonatal death, intrauterine growth restriction, perinatal death, preterm birth, preterm PROM, term PROM, maternal death, eclampsia, infant death congenital malformations, birth weight; no reported adverse effects.	High	Majority moderate-high quality but low for preterm PROM
			Impacted gestational age (MD 0.31, CI 0.01 to 0.61) and the risk of self-reported abdominal pain.		
			Risk of term and preterm PROM differed by type of supplementation–reduced risk for term and preterm PROM when supplemented alone, but increased risk when supplementation included vitamin C and E (RR 1.73, CI 1.34 to 2.23)		
***Rogne et al. (2017) [70]***	B12	Birth weight, LBW, SGA, preterm	No associations between B12 levels and birth weight but B12-deficiency was associated with an increased risk of LBW (RR1.15, CI 1.01 to 1.31) and higher B12 was associated with higher birth weight in LMIC. Linear association between maternal levels of B12 and risk of preterm birth (0.89 per one SD increase (CI 0.82 to 0.97).	High-moderate	Overall moderate-high quality
***Makrides et al. (2014) [71]***	Magnesium	Perinatal mortality, maternal mortality, pre-eclampsia, stillbirths, neonatal death prior to discharge, preterm, birth weight, LBW, gestational age	No impact perinatal mortality, SGA, stillbirth, LBW, pre-eclampsia; No data on maternal death; Increase in neonatal death (RR 2.21, CI 1.02–4.75); fewer babies with an Apgar score less (RR 0.34, CI 0.15 to 0.80).	High	Lack of high-quality evidence; selective reporting unclear for all trials; 20% of trials high risk due to rack of blinding of participants
***Chaffee et al. (2012) [72]***	Zinc	Preterm, LBW, SGA, birth weight, gestational age, head circumference	Impacted preterm (RR 0.86, CI 0.75 to 0.99); no effect on LBW, SGA, birth weight, gestational age, head circumference.	High	Overall very low-low
***Buppasiri et al. (2015) [73]***	Calcium	Preterm birth, LBW, maternal death, maternal weight, perinatal mortality, neonatal death, birth length, head circumference, intrauterine growth restrictions, mineral bone density	Impacted birth weight (MD 56.40g, CI 13.55 to 99.25g), and infant total and tibial bone mineral density.	High	Overall moderate quality
			No impact LBW, or preterm birth, maternal death, perinatal mortality, birth length, head circumference, intrauterine growth restrictions, maternal bone mineral density. No data on neonatal death.		
***Lassi et al. (2013) [74]***	Folic Acid	Preterm birth, stillbirths/neonatal death, pre-eclampsia, LBW, birth weight, maternal haemoglobin	No impact preterm, no impact on stillbirth/neonatal deaths, LBW and mean birth weight, maternal haemoglobin.	High	Included studies old (30 to 45 years ago); outcome measurement varied, poor compliance with random allocation, concealment and blinding. Bias and confounding seemed likely explanations for findings
***Vitamin D***					
***Bi et al. (2018) [75]***	Vitamin D	Infant growth, birth weight, LBW, morbidity, and mortality	Positive impact SGA (RR 0.72, CI 0.52 to 0.99), risk of foetal/neonatal mortality (RR 0.72, CI 0.47 to 1.11) or cognitive abnormalities (RR 0.94, CI 0.61 to 1.43), birth weight (MD, 75.38g, CI 22.88 to 127.88 g), and weight at 3, 6, 9, and 12 months. No significant difference in LBW or preterm birth.	High	Majority low/unclear risk of bias
***Perez-Lopez et al. (2015) [76]***	Vitamin D	Pre-eclampsia, SGA, LBW, preterm, birth weight, birth length	Impact on birth weight (MD 107.6g, CI 59.9 to 155.3g), and birth length (MD 0.3cm, CI 0.1 to 0.41cm). No impact on pre-eclampsia, SGA, LBW and preterm birth.	High	Majority unclear/low risk of bias
					Low risk of bias for all studies for incomplete data, selective reporting and other sources
***Gallo et al. (2020) [77]***	Vitamin D	Preeclampsia, SGA, birth weight and length	Increase in infant 25 (OH)D concentration, and birth weight (MD 114.2g, CI 63.4 to 165.1g). No impact on SGA and birth length or pre-eclampsia.	High	Overall fair quality
***Maugeri et al. (2019) [78]***	Vitamin D	LBW, SGA, birth weight, length, and head circumference	Positive impact birth weight (MD 103.17g, CI 62.29 to 122.04g), birth length (MD 0.50 cm, CI 0.08 to 0.92cm), head circumference (MD 0.19cm, CI 0.1 to 0.24cm), LBW (RR 0.40, CI 0.22 to 0.74), SGA (RR 0.69, CI 0.51 to 0.92); all outcomes impacted with Vit D supplemented alone, no impact when combined with other micronutrients.	High	Ranged very low (head circumference) to moderate quality (birth weight, birth length, LBW, and SGA)
***Lactation***					
***Ndikom, et al. (2014) [79]***	Extra fluids for breastfeeding mothers for increasing milk production.	Weight gain; EBF duration; Breastmilk production	No data on LMIC found despite inclusion in the search. Only one study met the inclusion criteria: reported no impact on breastmilk production (measured using test weighting) following advice to mothers to drink extra fluids. No data on other primary or secondary infant outcomes.	High-moderate	Low-quality
***Foong et al. (2020) [80]***	Oral galactagogues	Continued breastfeeding, infant weight, milk volume	**Pharmacological:** one study that reported on infant weight, little/no difference; possible effect on milk volume (MD 63.82 ml, CI 25.91 to 101.72ml).	High	Very low-low quality
			**Natural**: Moringa and mixed botanical tea may increase infant weight; little/no difference in milk volume.		
			Uncertain of adverse effect for mothers, those reported were minor complaints.		
***Campion-Smith et al. (2020) [7]***	MMN and polyunsaturated fatty acid supplementation.	Infant and child mortality, morbidity, and growth: weight, length, head circumference, WAZ, length-for-age z score	**Overall:** no impact infant and child mortality and morbidity	Moderate-low	Not available for all outcomes: Wide range very low-high
			Polyunsaturated fatty acid supplementation vs placebo: no impact infant length, weight and head circumference		
			Polyunsaturated fatty acid supplementation during gestation and lactation: no significant growth effects, some evidence for impact on child attention beyond 24 months of age.		
			An RCT of maternal calorie supplementation + breastfeeding support: no impact WAZ or length for age z score; Increased infant breast milk intake and EBF.		
***Abe et al. (2016) [81]***	MMN	Infant mortality, morbidity, adverse effects within three days of supplementation	No evidence to quantitatively assess the effectiveness of MMN supplementation in improving health outcomes in mother and baby.	High-moderate	Poorly reported among original studies; unclear risk of bias
			Impact on maternal anaemia (1 study) and vitamin B12 and folic acid milk concentration, but no significant dereferences were reported in serum concentrations (1 study).		
***Pregnancy and lactation***					
***Petry et al. (2016) [82]***	Low does iron and zinc via supplementation or fortification	Birth weight, LBW, HAZ, WAZ, WHZ, mental and motor development, morbidity.	**Zinc, pregnancy:** No impact on LBW, birth weight, one study found a beneficial effect of HAZ at 6months, no impact on other growth parameters. **Zinc, lactation:** One study (20mg, 40mg and control), no effect on breastmilk, growth or serum zinc concentration in exclusively breastfed infants.	High-moderate	Majority low-moderate quality
			**Iron, pregnancy**: No impact on birth weight, LBW, mental development scores; no data on child growth. **Iron, lactation**: No impact on birth weight, LBW, mental development scores; no data on child growth.		

Standard mean difference (SMD); Height-for-age z score (HAZ); Weight-for-age z score (WAZ); Mid-upper arm circumference (MUAC); Weight-for-height z scores (WHZ); Small for gestational age (SGA); Mean difference (MD); Relative risk (RR); Low birth weight (LBW); Randomised control trails (RCTs); Multiple micronutrients (MMN); **Premature rupture of membranes (PROM); Low middle-income countries (LMIC).**

*Quality reported in the respective review.

Reviews of MMN supplementation found that compared to no supplementation or supplementation with three or fewer micronutrients, they positively impacted risk of LBW and SGA [41, 62]. There was no statistically significant difference between supplements that contained at least 60 mg compared to supplement containing 30mg of iron and there was no difference in the timing of the intervention [62]. There was an increased risk of neonatal death in MMN intervention beginning after the first trimester when compared to iron folic acid supplementation, though this finding was based on low-quality evidence [62].

**Vitamin D** supplementation is a growing area of research; four reviews found a positive impact on birth weight, particularly if given after 20 weeks’ gestation, with potentially continued effect at 3, 6, 9 and 12 months [75–78]. Deficient women may benefit more [77]. Z**inc** supplementation has a positive effect on risk of preterm birth but no evidence for other infant outcomes [28, 72, 82]. Iron folic acid supplementation was associated with increased birth weight [28, 41]. **Iodine supplementation** may improve growth outcomes in severely deficient women [28, 65–67]. When stratified by country income there was an association between vitamin B12 and birth weight in LMICs, but not in HICs [70]. There is mixed evidence of the effect of **omega-three fatty acid supplementation on birth outcomes [64]. There was also mixed evidence for calcium supplementation on birth outcomes [28, 73].** It may be beneficial in low intake populations, or if given after 20 weeks of gestation **[28, 73]**. Cochrane reviews of vitamin A, E and C supplementation showed no clinically significant impact on infant outcomes [63, 68, 69]. There is no quality evidence on effect of magnesium supplementation on birth outcomes [71].

#### Maternal supplementation during lactation

We found only five reviews examining the role of supplementation during lactation [7, 79–82]. There was no conclusive evidence that zinc supplementation, polyunsaturated fatty acid supplementation nor MMN provided to breastfeeding mothers impacted infant health and growth outcomes [82]. A review of galactagogues found some impact of Moringa and mixed botanical galactagogues on infant weight [7, 80] (Table 2). One Cochrane review explored extra fluids for breastfeeding mothers, with the rationale that this might increase breastmilk production [79]. Authors concluded that there was “Not enough evidence to increase fluid intake beyond what likely to require for comfort” [79].

## Discussion

Our review found a large number of good quality reviews which explore maternal-focused interventions to prevent or address growth faltering in infants <6m. Evidence from these can inform future management strategies for small and nutritionally at-risk infants <6m in LMICs. The options for maternal interventions include a wide range of interventions as reflected in our results categorisation: breastfeeding promotion interventions; education and counselling; WASH; women’s empowerment; relaxation therapies; maternal mental health; m-health; conditional cash transfers; agriculture, maternal supplementation in pregnancy; maternal supplementation during lactation; and multi-sectoral interventions.

Breastfeeding promotion interventions during pregnancy and promotion and counselling during lactation are effective at improving infant feeding practices however evidence is more limited on anthropometric impacts. Outcomes relating to infant feeding practices are generally self-reported and not objectively measured [83]. More research is needed on community-based breastfeeding education and counselling that effectively improve growth outcomes in small or nutritionally at-risk infants <6m. Most evidence is for prevention of growth faltering, however there is evidence that lactation support can be used to successfully manage growth faltering at birth [84–86], and in older infants [87, 88]. In the documented experiences of implementing a “MAMI” approach (Management of small and nutritionally at-risk infants under six months) in an inpatient setting, most infants presenting with growth faltering had the possibility to breastfeed and tailored, structured lactation counselling was successfully used to establish EBF and secure weight gain in most infants [87–89]. However, there were considerable challenges in achieving this for some higher risk infants and sustaining progress post-discharge in the absence of community follow-up [87]. Educating family members, such as fathers and mothers-in-laws, has also shown a positive effect on neonatal mortality and feeding practices, emphasizing the importance of an inclusive approach and considering the wider family dynamics that influence feeding practices [37, 90]. Breastfeeding support packages in the workplace have shown positive impacts on breastfeeding initiation and duration. In high income settings they have also been found to be feasible to implement, presenting an opportunity to further target and support mothers and their infants [91]. However, this is much less evidenced in LMICs that also need to consider the informal work settings that so many mothers worldwide engage in. Education and counselling interventions, such as those focused on complementary feeding or care practices, generally have a positive but modest effect on infant growth outcomes; as with any education intervention, the effectiveness is likely to be proportional to the quality, content and uptake of the education provided [92]. Education interventions in combination with food supplementation or conditional cash transfers appear to be more impactful. Guidance on what constitutes effective education and counselling interventions for improving infant growth would be valuable for programmers faced with managing small and nutritionally at-risk infants <6m.

WASH interventions had some positive effects on linear growth, although less so for infants <6m. Women’s empowerment and m-health interventions were also potentially effective when combined with agriculture interventions or nutrition-specific interventions such as micronutrient supplementation. One possible mechanism behind these interventions is that maternal diet is improved, either during pregnancy or lactation, which in turn supports better in utero/infant growth and nutrition. The rationale behind conditional cash transfer programmes is similar and there is evidence of some positive impact on infant growth outcomes, however the evidence notes an important potential side-effect, which is the increased maternal consumption of high-fat or high-sugar foods in middle income settings. While low maternal body mass index and micronutrient deficiencies in pregnancy and during lactation are associated with infant growth faltering, so is high body mass index [2, 93–95].

Maternal mental health is perhaps one of the most promising but under-researched areas for post-natal intervention. Evidence for the association between maternal mental health and infant growth is strong; a systematic review found postnatal depression was associated with underweight in infants <6m in two studies and poor weight and height in a third, and is linked to poor breastfeeding practice and increased illnesses in other studies [96]. Alongside maternal depression, increased stress biomarkers are associated with intrauterine growth restrictions and poor growth through the post-natal period [97]. A meta-analysis of cohort studies found maternal anxiety during pregnancy was associated with significant increased risk of pre-term birth and LBW [98]. A meta-analysis estimated that 23% to 29% fewer children would be underweight or stunted if the infant population were unexposed to maternal depressive symptoms [99]. Despite strong associations, we found few reviews on maternal mental health interventions for infant growth and nutrition. One review found that cognitive behavioural therapy during pregnancy had no impact on infant weight or growth, but a positive impact on depression post-partum. However, Fotiou et al (2017) found that cognitive behavioural counselling had a positive impact on breastfeeding initiation and the duration of EBF [100]. In addition, there is promising evidence from relaxation therapy while breastfeeding, which has been found to reduce maternal stress levels, increased milk yield and milk fat levels, especially in mothers of preterm infants. Since that review was published, a randomised controlled trial (RCT) found greater weight gain and body mass index in infants in the relaxation intervention group but no effect on head circumference and length [101]. They also found a significant effect on the duration of sleep at 6–8 weeks and reduced cortisol concentrations in breastmilk [101]. Relaxation therapy is a fast-evolving area of research [102, 103]; other context-specific interventions that relieve maternal stress while breastfeeding could have similar effects.

Lastly, interventions to supplement maternal diets with macronutrients, MMN, vitamin D, zinc, iron folic acid, and possibly calcium, iodine and B12 in deficient women, in pregnancy, can improve birth outcomes. However, a recent RCT, not captured by the reviews, found that ready-to-use supplementary food plus daily MMN antenatal supplementation for moderately malnourished pregnant women was only modestly effective at improving length at birth, 6 weeks and 12 weeks, despite the large quantity of calories provided; this suggests that in high-risk settings stunting in utero is unlikely to be reduced by supplementation alone [104]. While there is evidence that low postnatal maternal body mass index is a risk factor for infant growth faltering, there is currently little evidence for the effect of maternal supplementation during lactation to improve infant growth. There were also few intervention studies for improving maternal dietary diversity during pregnancy and lactation, a known risk factor for poor infant growth outcomes [105]. Interventions seeking to address maternal dietary diversity could also influence infant diets during complementary feeding after six months age, another known risk factor for poor later child growth [106].

### Limitations

We acknowledge several limitations to our review. First, limiting our search to reviews means that some primary evidence may have been missed. On the other hand, this approach also enabled us to capture a broader range of interventions and set the stage for important future research.

We also acknowledge that we only included reviews published since 2008. This is unlikely however to have altered our overall conclusions since individual reviews still include earlier original studies. Another limitation is that the same primary studies could have been included in several reviews; this may have given unwarranted weighting of the evidence from one study included across multiple reviews. Furthermore, while all reviews included studies from LMIC, several reviews (61%) also included studies from HIC; the inclusion of HIC may affect the applicability of our results to LMIC settings.

We were constrained in our ability to disaggregate data depending on how data was reported. Many reviews did not disaggregate data on infants <6m from older children; recognising infants <6m as an important distinct age category is vital to build evidence for best practice. Similarly, some reviews included studies that combined the exposure of interest with supplementation or included studies with special populations, such as those at increased risk due to underlying conditions. This may have affected the comparability of results but due to the small number of these studies, we do not believe this significantly affected our study’s findings. Variability across the infant outcomes measured made comparing and quantifying the effects of different interventions difficult. Whilst the quality of the reviews was generally high, the quality of the respective primary studies was sometimes low. GRADE criteria were used to assess the quality of the included reviews; while it is a standardised and widely recognised method, it does not account for the inclusion of grey literature, which can increase the quality of reviews. There were also several interventions with only very limited evidence, such as a small number of studies from a limited number of contexts, not RCTs, and small sample sizes.

Most evidence we found relates to the prevention of growth faltering, rather than the management of already small and nutritionally compromised infants <6m. This highlights the need for future studies to focus specifically on this group; whilst it is plausible and likely that interventions which work for prevention also work for management, it is not certain and effect sizes may differ. Most studies were also timed around the antenatal rather than the postnatal period. Future programmes should support mothers throughout (and before) pregnancy but better understanding of the relative impact of different interventions at different times is still important to help prioritise and target. Whilst this review focused only on outcomes for the infant, outcomes for the mother are equally important and also warrant attention and action.

### Policy implications

The heterogeneity across the zero to six month period, transitioning from the umbilical cord to solid feeding, and the contextual dynamic and needs of the individual mother-infant dyad, will likely warrant an assortment of both maternal and infant-focused interventions, working together in synergy. While a coordinated strategy is required, one approach or set of interventions alone is unlikely to suit all contexts and all infants <6m. Addressing maternal as well as infant needs reflects a recent WHO report on Essential Nutrition Actions and the 2021 Lancet Maternal and Child Undernutrition series, recognising the importance of addressing nutrition throughout the life course [17, 107]. The UN ‘Global Action Plan’ (GAP) on Child Wasting: Framework for Action also stresses the importance of maternal nutrition interventions during the preconception, pregnancy and postnatal period, to improve infant outcomes, specifically for the reduction of LBW [108]. The need for essential nutrition packages to be integrated into national health systems and policies is also highlighted [108]; essential nutrition packages must include multifaced maternal interventions for managing small and nutritionally at-risk infant <6m. This could be achieved though strengthening referral pathways between services that are already available (e.g. maternal mental health, infant and young child feeding) or through the adoption of adaptable community-based interventions that combine maternal and infant nutrition to fill service gaps. One example of such an approach is a recently released integrated care pathway for managing small and nutritionally at-risk infants <6m and their mothers [109].

## Conclusion

Our review of reviews identifies a variety of interventions aimed at mothers in the antenatal and postnatal period in LMIC that could be used to also improve infant nutrition outcomes. Several interventions proved to be effective in preventing growth faltering in infants <6m, including: breastfeeding, education and peer support, counselling interventions, antenatal supplementation combined with women’s empowerment, m-health technologies, WASH, conditional cash transfers, and/or agriculture interventions. Relaxation therapies and mental health interventions also have potential–but more evidence is needed. Besides galactagogues, evidence on post-natal maternal supplementation for managing infant growth faltering has shown limited impact, although the number of studies is limited. There is limited evidence that directly considers impact of interventions on small and nutritionally at-risk infants <6m, highlighting an important research gap. Based on this collated evidence, more holistic packages of care that include maternal interventions are justified; contextualised support of the mother-infant dyad could help small and nutritionally vulnerable infants aged <6m survive and thrive.

## Supporting information

S1 Table Search strategy (MEDLINE format). (DOCX)

S2 Table GRADE quality assessment for maternal non-supplementation interventions during lactation and pregnancy–reviews. (DOCX)

S3 Table GRADE quality assessment for maternal supplementation interventions during lactation and pregnancy–reviews. (DOCX)

S4 Table PRISMA checklist. (DOCX)

## List of abbreviations

<6m: Less than 6 months
EBF: Exclusive breastfeeding
GRADE: Grading of Recommendations, Assessment, Development and Evaluations
HAZ: Height-for-age z‐score
LBW: Low birth weight
LMIC: Low -and middle- income countries
MD: Mean difference
MMN: Multiple micronutrient
MUAC: Middle-upper-arm-circumference
RCT: Randomised control trial
RR: Relative Risk
SGA: Small for gestational age
WASH: Water Sanitation and Hygiene
WAZ: Weight‐for‐age z-score
WHZ: Weight‐for‐height z‐score
