# Supplementary material for: Maternal-focused interventions to improve infant growth and nutritional status in low-middle income countries: A systematic review of reviews
Source: PLoS One. 2021 Aug 18;16(8):e0256188. doi: 10.1371/journal.pone.0256188 (PMC8372927; doi:10.1371/journal.pone.0256188)
Supplement: S1 Table — (DOCX) [file pone.0256188.s001.docx]

## **S1 Table.** Search strategy (MEDLINE format)

|  | Search Terms |
| --- | --- |
| Concept 1 Mothers | pregnan* OR “pregnant wom?n” OR maternal OR mother*  exp Mothers/  exp pregnant women/  pregnancy/ |
| Concept 2 Possible Interventions | intervention* OR nutrition OR “nutrition-specific intervention*” OR “nutrition specific intervention*” OR supplementation OR supplem* OR “food distribution” OR “nutritional counsel?ing” OR macronutrients OR macronutr* OR “nutrition micronutrients” OR micronutrients OR micronutr* OR “food fortification” OR “multi-micronutrient sprinkles” OR iron OR “folic acid” OR “nutrition-sensitive intervention*” OR “nutrition sensitive intervention*” OR employment OR workplace OR income OR “cash transfers” OR “financial incen*” OR “women empowerment” OR “wom?n group*” OR PLA OR “sanitation & water” OR “water supply” OR “water source” OR latrine OR “pit latrine” OR toilet OR hygiene OR WASH OR “wat* san*” OR drainage OR “hand washing” OR handwashing OR “hand hygiene” OR soap OR detergent OR “hygiene promotion” OR “open defecation” OR “maternal mental health” OR “mental health” OR depression OR depress* OR anxiety OR exercise OR wellbeing OR lifestyle OR relaxation OR “relaxation therapy” OR hydration OR hydr* OR “hydration therapy” OR violence OR “domestic violence” OR “gender based violence” OR “food insecurity”  exp Hand Hygiene/  exp Mental health/ |
| Concept 3 Outcomes | infan* OR baby OR babies OR post-natal OR postnatal OR neonate OR “neonatal mortality” OR “infant mortality” OR “neonatal death” OR morbidity OR “nutritional status” OR growth OR “infant growth” OR “failure to thrive” OR FTT OR “growth failure” OR “growth disorder” OR malnutr* OR malnourish* OR “acute malnutrition” OR undernourish* OR undernutr* OR stunt* OR wasting OR underweight* OR “feeding practices” OR “low weight-for-length” OR weight-for-length OR WFL OR WLZ OR “low weight-for-age” OR weight-for-age OR WFA OR WAZ OR “low length for age” OR “length for age” OR LFA OR LAZ OR anthropometry OR “anthropometric indicators” OR MUAC OR “mid upper arm circumference” OR “low MUAC” OR “low birth weight” OR LBW OR “birth weight” OR breastfeeding OR “breast feeding” OR breastfeed* OR lactation OR IYCF OR “infant and young child feeding” OR deficiency OR deficen* OR “folic acid deficiency” OR anaemia OR anemia OR haemoglobin OR haemoglobin  exp Malnutrition/  exp Breast Feeding/  exp Nutritional Status |
| Concept 4 Setting | MEDLINE all country designated as LMIC by World Bank 2008 |
| Concept 1+2+3+4 |  |
| **+ Limitation review + human, since 2009** |  |
