# Supplementary material for: Maternal-focused interventions to improve infant growth and nutritional status in low-middle income countries: A systematic review of reviews
Source: PLoS One. 2021 Aug 18;16(8):e0256188. doi: 10.1371/journal.pone.0256188 (PMC8372927; doi:10.1371/journal.pone.0256188)
Supplement: S2 Table — (DOCX) [file pone.0256188.s002.docx]

## **S2 Table.** GRADE Quality assessment for maternal non-supplementation interventions during lactation and pregnancy – reviews.

| **Criteria**  **(yes/no/not clear)** | **Review articles** | | | | | | | | | | | | | | | | |
| --- | --- | --- | --- | --- | --- | --- | --- | --- | --- | --- | --- | --- | --- | --- | --- | --- | --- |
|  | Arikpo (2018) | Bhutta (2013) | Goudet (2019) | Kraft (2014) | Gizaw (2019) | Dangour (2013) | Ota (2015) | Ruel (2013) | Saronga (2019) | Shakya (2017) | Taukobong (2016) | Victora (2012) | Gera (2018) | Giugliani (2015) | Leroy (2009) | Lagarde (2009) | Masset (2012) |
| Does the SR explicitly report and perform a comprehensive and reproducible literature search? | yes | yes | yes | no | yes | yes | yes | yes | yes | yes | yes | No | no | yes | no | yes | no |
| Does the SR formulate clearly focused questions? | yes | yes | yes | yes | yes | yes | yes | yes | yes | yes | yes | yes | yes | yes | yes | yes | yes |
| Does the SR's methods section explicitly state the basis for inclusion or exclusion of primary RCT's? | yes | not clear | yes | yes/no | yes | yes | yes | not only RCTs | yes | yes | yes/no | Yes/no | Yes/no | no | no | yes | yes |
| Does the SR report data from primary RCT's (e.g. size, interventions used, results from individual RCT's | yes | yes | yes | yes | yes | yes | yes | no | yes | yes | yes | yes | yes | yes | yes | yes | yes |
| Does the SR assess the methodological quality of primary studies, and take these into account when necessary? | yes | no | yes | no | yes | yes | yes | no | yes | yes | Not formally | no | yes | yes | no | yes | yes |
| Meta-analysis: does the SR combine primary studies appropriately? | yes | N/A | Yes | N/A | yes | yes | yes | N/A | N/A | yes | N/A | N/A | yes | yes | N/A | N/A | Yes/no (only for serum retinol conc) |
| Meta-analysis: does the SR state how results are combined statistically? | yes | N/A | Yes | N/A | yes | yes | yes | N/A | N/A | yes | N/A | N/A | yes | yes | N/A | N/A | yes |
| Meta-analysis: does the SR report absolute numbers as well as appropriate summary statistics? | yes | N/A | yes | N/A | yes | yes | yes | N/A | N/A | yes | N/A | N/A | yes | yes | N/A | N/A | yes |
| Does the SR report on clinical relevance/importance of the results? | yes | yes | yes | yes | yes | yes | yes | yes | yes | yes | yes | yes | yes | yes | yes | yes | yes |
| **Overall quality of review** | **High** | **High-moderate** (captured only reviews with good quality methodologies, spanned wide range of topics, comprehensive review) | **High** | **Moderate-low** (One reviewer screened) | **High** | **High** | **High** | **Moderate** | **High-moderate** (two reviewers, low number of studies, included RCT, pre/post and quasi-experimental, English only, no grey lit) | **High** | **Moderate** (screened by interdisciplinary team) | **Low** (case studies appear to be picked to give example of success and failure, rather than all literature equally evaluated) | **Moderate** | **High-moderate** | **Moderate-low** | **High** (include grey literature, two reviewers, comprehensive review, no language restrictions) | **Moderate** |

| **GRADE Quality assessment for maternal non-supplementation interventions during lactation and pregnancy – reviews (continued)** | | | | | | | | | | |
| --- | --- | --- | --- | --- | --- | --- | --- | --- | --- | --- |
| **Criteria**  **(yes/no/not clear)** | **Review articles** | | | | | | | | | |
|  | Lassi (2020) | Jonmohamed (2020) | Lassi (2019) | Ojha (2020) | Palmer (2020) | Sondaal (2016) | Park (2019) | Park (2020) | East (2019) | Shukir (2018) |
| Does the SR explicitly report and perform a comprehensive and reproducible literature search? | yes | yes | yes | yes | yes | yes | yes | yes | yes | yes |
| Does the SR formulate clearly focused questions? | yes | yes | yes | yes | yes | yes | yes | yes | yes | yes |
| Does the SR's methods section explicitly state the basis for inclusion or exclusion of primary RCT's? | yes | yes | yes | yes | yes | yes | yes | yes | yes | yes |
| Does the SR report data from primary RCT's (e.g. size, interventions used, results from individual RCT's | yes | yes | yes | yes | yes | yes | yes | yes | yes | yes |
| Does the SR assess the methodological quality of primary studies, and take these into account when necessary? | yes | yes | yes | yes | yes | yes | yes | yes | yes | yes |
| Meta-analysis: does the SR combine primary studies appropriately? | yes | yes | yes | yes | yes | NA | yes | yes | yes | N/A |
| Meta-analysis: does the SR state how results are combined statistically? | yes | yes | yes | yes | yes | NA | yes | yes | yes | N/A |
| Meta-analysis: does the SR report absolute numbers as well as appropriate summary statistics? | yes | yes | yes | yes | yes | NA | yes | yes | yes | N/A |
| Does the SR report on clinical relevance/importance of the results? | yes | yes | yes | yes | yes | yes | yes | yes | yes | yes |
| **Overall quality of review** | **High** | **High** | **High** | **High** | **High** | **High-moderate** (inclusion of grey literature, meta-analysis not possible due to heterogeneity, data extracted from database one reviewer) | **High** | **High** | **High** | **High-moderate** (few studies, excluded observational but reason given, limited to English) |
