# Supplementary material for: Maternal-focused interventions to improve infant growth and nutritional status in low-middle income countries: A systematic review of reviews
Source: PLoS One. 2021 Aug 18;16(8):e0256188. doi: 10.1371/journal.pone.0256188 (PMC8372927; doi:10.1371/journal.pone.0256188)
Supplement: S3 Table — (DOCX) [file pone.0256188.s003.docx]

## **S3 Table**. GRADE Quality assessment for maternal supplementation interventions during lactation and pregnancy – reviews

| **Criteria**  **(yes/no/not clear)** | **Review articles** | | | | | | | | | | | | | | | | |
| --- | --- | --- | --- | --- | --- | --- | --- | --- | --- | --- | --- | --- | --- | --- | --- | --- | --- |
|  | Fong (2020) | Petry (2016) | Campion-Smith (2020) | Abe (2016) | Gresham (2014) | Visser (2018) | Stevens (2015) | Imdad (2011) | Pimpin (2019) | Ramakrishnan (2012) | Throne-Lyman (2012) | Das (2018) | Middelton (2018) | Harding (2017) | Farebrother (2018) | Zhou (2013) | Rumbold (2015)a |
| Does the SR explicitly report and perform a comprehensive and reproducible literature search? | yes | yes | Not clear | yes | Yes/no (referenced corresponding study) | yes | yes | yes | yes | Yes | Yes | Yes | Yes | Yes | yes | Yes | Yes |
| Does the SR formulate clearly focused questions? | yes | yes | yes | yes | yes | yes | yes | yes | yes | yes | Yes | Yes | Yes | Yes | yes | Yes | Yes |
| Does the SR's methods section explicitly state the basis for inclusion or exclusion of primary RCT's? | yes | yes | yes | yes | yes | yes | yes | yes | yes | yes | Yes | Yes | Yes | Yes | yes | Yes/no | Yes |
| Does the SR report data from primary RCT's (e.g. size, interventions used, results from individual RCT's | yes | Yes/no | no | yes | yes | yes | yes | yes | yes | yes | Yes | Yes | Yes | Yes | yes | Yes | Yes |
| Does the SR assess the methodological quality of primary studies, and take these into account when necessary? | yes | yes | Yes/no (not for all papers, not overall) | yes | Yes | yes | yes | yes | yes | yes | Yes | Yes | yes | Yes | yes | yes | Yes |
| Meta-analysis: does the SR combine primary studies appropriately? | yes | yes | NA | NA | yes | yes | yes | yes | Yes | yes | Yes | Yes | Yes | Yes | Yes | NA | Yes |
| Meta-analysis: does the SR state how results are combined statistically? | yes | yes | NA | NA | yes | Yes | yes | yes | yes | yes | Yes | Yes | yes | Yes | Yes | NA | Yes |
| Meta-analysis: does the SR report absolute numbers as well as appropriate summary statistics? | yes | yes | NA | NA | yes | Yes | yes | yes | Yes | yes | Yes | Yes | Yes | Yes | Yes | NA | Yes |
| Does the SR report on clinical relevance/importance of the results? | yes | yes | yes | yes | yes | yes | yes | yes | yes | Yes | Yes | yes | yes | Yes | yes | yes | yes |
| **Overall quality of the review** | **High** | **High-moderate** (unclear if two reviewers) | **Moderate-low** (one reviewer screened, due to scope a full systematic search strategy was not implemented) | **High-moderate** (only two studies, two reviewer’s comprehensive method, but limited to RCTs) | **High** (two reviewers**)** | **High** | **High** | **High** | **High** | **High** | **High-moderate** (one reviewer screened) | **High** | **High** | **High** | **High** | **High-moderate** (unable conduct met-analysis due to data scarcity, no language limits, limited to RCT’s) | **High** |

| **GRADE Quality assessment for maternal supplementation interventions during lactation and pregnancy – reviews (continued)** | | | | | | | | | | | |
| --- | --- | --- | --- | --- | --- | --- | --- | --- | --- | --- | --- |
| **Criteria**  **(yes/no/not clear)** | **Review articles** | | | | | | | | | | |
|  | Rogne (2017) | Makrides (2014) | Chaffee (2012) | Buppasiri (2015) | Lassi (2013) | Bi (2018) | Perez-Lopes (2014) | Gallo (2019) | Mugeri (2019) | Ndikom (2014) | Rumbold (2015)b |
| Does the SR explicitly report and perform a comprehensive and reproducible literature search? | yes | yes | yes | Yes | yes | yes | yes | yes | Yes | Yes | Yes |
| Does the SR formulate clearly focused questions? | yes | yes | Yes | yes | yes | yes | yes | yes | yes | Yes | Yes |
| Does the SR's methods section explicitly state the basis for inclusion or exclusion of primary RCT's? | yes | yes | yes | yes | yes | yes | yes | yes | yes | Yes | Yes |
| Does the SR report data from primary RCT's (e.g. size, interventions used, results from individual RCT's | yes | yes | yes | yes | yes | yes | yes | yes | yes | Yes | Yes |
| Does the SR assess the methodological quality of primary studies, and take these into account when necessary? | yes | yes | yes | Yes | yes | yes | yes | yes | yes | Yes | Yes |
| Meta-analysis: does the SR combine primary studies appropriately? | yes | yes | yes | yes | yes | yes | yes | Yes | yes | NA | Yes |
| Meta-analysis: does the SR state how results are combined statistically? | yes | yes | yes | yes | Yes | yes | yes | yes | yes | NA | Yes |
| Meta-analysis: does the SR report absolute numbers as well as appropriate summary statistics? | yes | yes | yes | yes | Yes | yes | yes | Yes | yes | NA | Yes |
| Does the SR report on clinical relevance/importance of the results? | yes | yes | yes | yes | yes | yes | yes | yes | Yes | Yes | yes |
| **The overall quality of the review** | **High-moderate** (screened by one author but the risk of bias assessed by two authors) | **High** | **High** | **High** | **High** | **High** | **High** | **High** | **High** | **High-moderate** (only one study, cross-over studies excluded, no grey literature) | **High** |
